# Osmomixotrophy in Phytoplankton: Analysis of 46 Strains From Marine and Freshwater Habitats Indicates Prevalent Use of Organic Compounds

**DOI:** 10.1111/1758-2229.70420

**Published:** 2026-09-23

**Authors:** Nele Martens, Luisa Listmann, Jette Ludewigs, C.‐Elisa Schaum

**Affiliations:** ^1^ Institute of Marine Ecosystem and Fishery Science University of Hamburg Hamburg Germany

**Keywords:** DOM, mixotrophy, osmotrophy, *Ostreococcus*, picophytoplankton

## Abstract

Osmomixotrophic use of dissolved organic compounds may be more widespread than previously recognized, but research seems so far isolated and mostly focusing on single phytoplankton groups or strains. Here we combined data from 24 marine and 22 freshwater strains to analyse their ability to use dissolved organic compounds. The results emphasize that osmomixotrophy is ubiquitous in phytoplankton across functional groups and taxa isolated from various habitats and not strictly dependent on light or nutrient deficiencies. Differences found in phytoplankton originating from different habitats may result from adaptive responses to habitat conditions, but conclusions need to be drawn carefully in line with the limited number of taxa included per habitat and potential niche partitioning. This study underscores the ecological significance of heterogeneous nutritional strategies and the necessity to address existing knowledge gaps to finally draw conclusions about the impact of osmomixotrophy in the context of single ecosystems and on global scales.

## Graphical abstract

Analysing thousands of data points across 46 marine and freshwater strains, this study underscores that osmomixotrophy is a common, rather than exceptional, metabolic strategy in phytoplankton. These findings challenge the autotrophic paradigm, demonstrating that organic matter acquisition is a default state with significant implications for our understanding of global carbon cycles (96‐well plate: public domain/CC0; map generated in R).

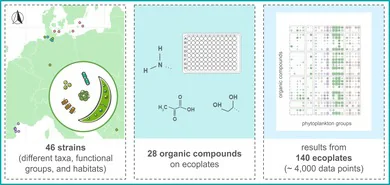

## 1 Introduction

Phytoplankton are still mostly considered autotrophs. However, recent studies challenge our understanding of the boundaries between different nutritional strategies among small aquatic organisms: Many phytoplankton taxa from various ecosystems have been shown to make use of organic material either by phagotrophy (Ahn and Glibert 2024; Costa et al. 2025; Millette et al. 2023) or by using dissolved organic compounds such as sugars (Godrijan et al. 2022; Liu et al. 2024; Villanova and Spetea 2021). The latter, also called osmomixotrophy, may be specifically widespread as it is not limited to certain morphotypes such as flagellates.

The use of organic matter affects the functioning of phytoplankton in ecosystems. For instance, exploiting osmoheterotrophic pathways can help phytoplankton to survive and grow under nutrient‐limited conditions (Foresi et al. 2022; Li et al. 2021; Zhang et al. 2021) and hence drive photosynthesis and CO_2_ fixation. Phagomixotrophic behaviour has been shown to increase the transfer efficiency along food webs (Ward and Follows 2016), but can also be linked to toxin production in response to inorganic nutrient depletion (Otte et al. 2025). Apart from a few studies that largely deal with phagotrophy (Ahn and Glibert 2024; Chen et al. 2024; Honig et al. 2025; Reinl et al. 2022; Wieczynski et al. 2023), the extent to which mixotrophic behaviour affects communities, ecosystems and substrate cycles—also in the context of climate change—remains widely unexplored.

Phytoplankton use organic compounds with the aim of obtaining energy, harvesting N and P, or to directly use the compounds in other ways (e.g., to build proteins). As phytoplankton are able to synthesize required materials themselves, it has been suggested that osmomixotrophy is a strategy to deal with conditions impeding photosynthetic growth, such as light, nutrient, or even CO_2_ limitation (Foresi et al. 2022; Jones et al. 2009; Li et al. 2021; Tuchman et al. 2006; Zhang et al. 2021). Yet, studies on phytoplankton's osmomixotrophic behaviour are often narrow, focusing on single compounds and strains covering single taxonomic groups and habitats.

Here we combined ecoplate data from 24 marine and 22 freshwater strains to analyse their ability to use 28 different dissolved organic compounds. Specifically, we wanted to compare the results among taxa and ecosystems.

## 2 Methods

### 2.1 Datasets and Study Areas

**FIGURE 1:**
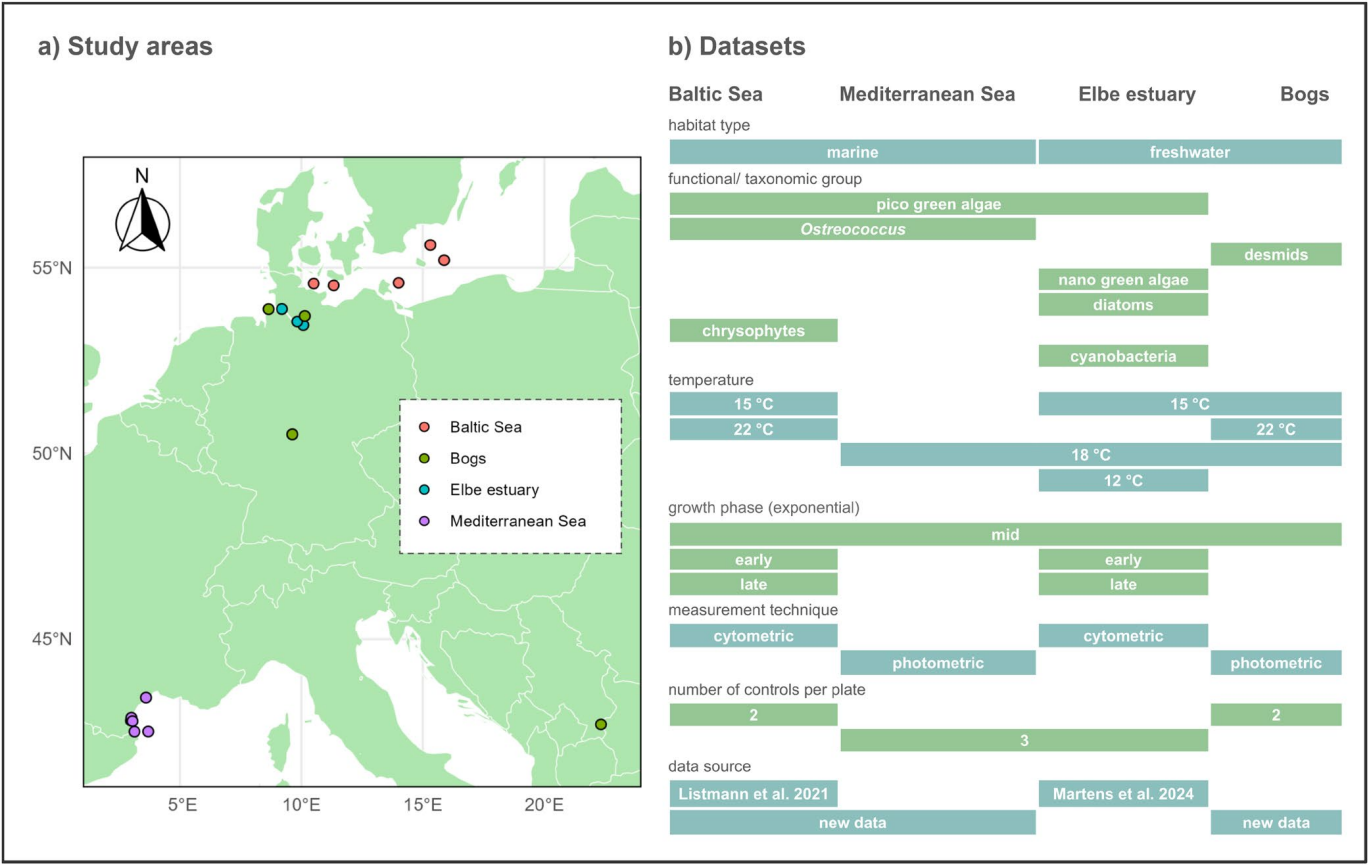
Overview of the included datasets: (a) shows the sampling stations from which the phytoplankton strains were isolated, (b) indicates where datasets differ and overlap with respect to additional parameters. Datasets are obtained from Listmann et al. (2021) and Martens et al. (2024) and previously unpublished data. See also Table S1. Map generated in R.

Our analysis comprises data from 140 ecoplates (see detailed description in Section 2.2) covering 46 phytoplankton strains (Table S1) from different study areas. Those include the Baltic Sea, the Mediterranean Sea, the Elbe estuary and different European bogs (Figure 1a, Table S1).

The Baltic Sea dataset is from previously published data (Listmann et al. 2021) and additional previously unpublished data. Chrysophytes and pico green algae (Chlorophyta < 3 μm)—including *Ostreococcus tauri* and *Ostreococcus mediterraneus*—were isolated from the Kiel area, Arkona Basin and Bornholm Basin (Figure 1a, Table S1). The Mediterranean Sea dataset contains new, previously unpublished data. Strains of *O. tauri* and *O. mediterraneus* were isolated from different lagoons, coastal areas and one open ocean station in the Gulf of Lion (Figure 1a, Table S1). Strains were provided by the group of Gwenael Piganeau (Evolutionary and Environmental Genomics, Observatoire Océanologique de Banyuls‐sur‐Mer). The Elbe estuary dataset has been published in (Martens et al. 2024). Strains were mainly isolated from the area around Hamburg and a few from further downstream (Figure 1a, Table S1). Strains include pico green algae (Chlorophyta < 3 μm) and nano green algae (Chlorophyta ~3–20 μm), cyanobacteria and a diatom (Bacillariophyta). Because the sampling sites are connected by continuous water masses, and to avoid further complexity, we do not distinguish exact sampling locations (Figure 1a) within the Baltic Sea, Mediterranean Sea and Elbe estuary in our analyses. The bog dataset contains new, previously unpublished data. It includes data of two desmids (Desmidiales; *Cosmarium botrytis* and *Closterium moniliferum* ). To avoid confusion between genera sharing the same initial, their names are not abbreviated. Strains were provided by the Microalgae and Zygnematophyceae culture collection of the University of Hamburg (MZCH) (Von Schwartzenberg et al. 2013). They were isolated from different bogs in Germany and Serbia (Figure 1a, Table S1) which we treat as separate habitats.

The datasets partially differ in terms of experimental design and other factors. This is because the data were obtained with different objectives and not originally designed to be compiled. Figure 1b shows where datasets overlap with respect to certain aspects and where they differ (see also Table S1). Each dataset contains a mostly unique set of taxa and functional groups (Litchman et al. 2007) (Figure 1b, Table S1, Figures 3 and 4). However, the group of pico green algae (here Chlorophyta < 3 μm) was included in the Baltic Sea, Mediterranean Sea and Elbe dataset. Additionally, multiple strains of *O. tauri* and *O. mediterraneus* were included in both the Mediterranean Sea and Baltic Sea dataset (Figure 1b). Also, among the bog dataset, *C. botrytis* and *C. moniliferum* were each isolated from at least two different bogs (Table S1, Figures 3 and 4). The present analysis comprises a total of 17 different taxa, which are not shown in detail in Figure 1b for clarity (see Table S1, Figures 3 and 4). Note that depending on the level of taxonomic resolution, these can be species (e.g., *O. tauri*), genera (e.g., *Mychonastes*) and in a few cases broader taxonomic groups (e.g., Synechococcales, unclassified pico green algae). Elbe and Baltic Sea strains were assessed at different time points during the exponential growth phase. Mediterranean Sea and bog strains were always assessed during the mid exponential phase (Figure 1b). Bog and Baltic Sea strains were assessed at different (treatment) temperatures (Table S1). Mediterranean Sea and Elbe strains were always assessed at their incubation temperature (Figure 1b, Table S1). Further, the measurement techniques differed for the datasets (Figure 1b, see also Table S1).

### 2.2 Laboratory Procedures

**FIGURE 2:**
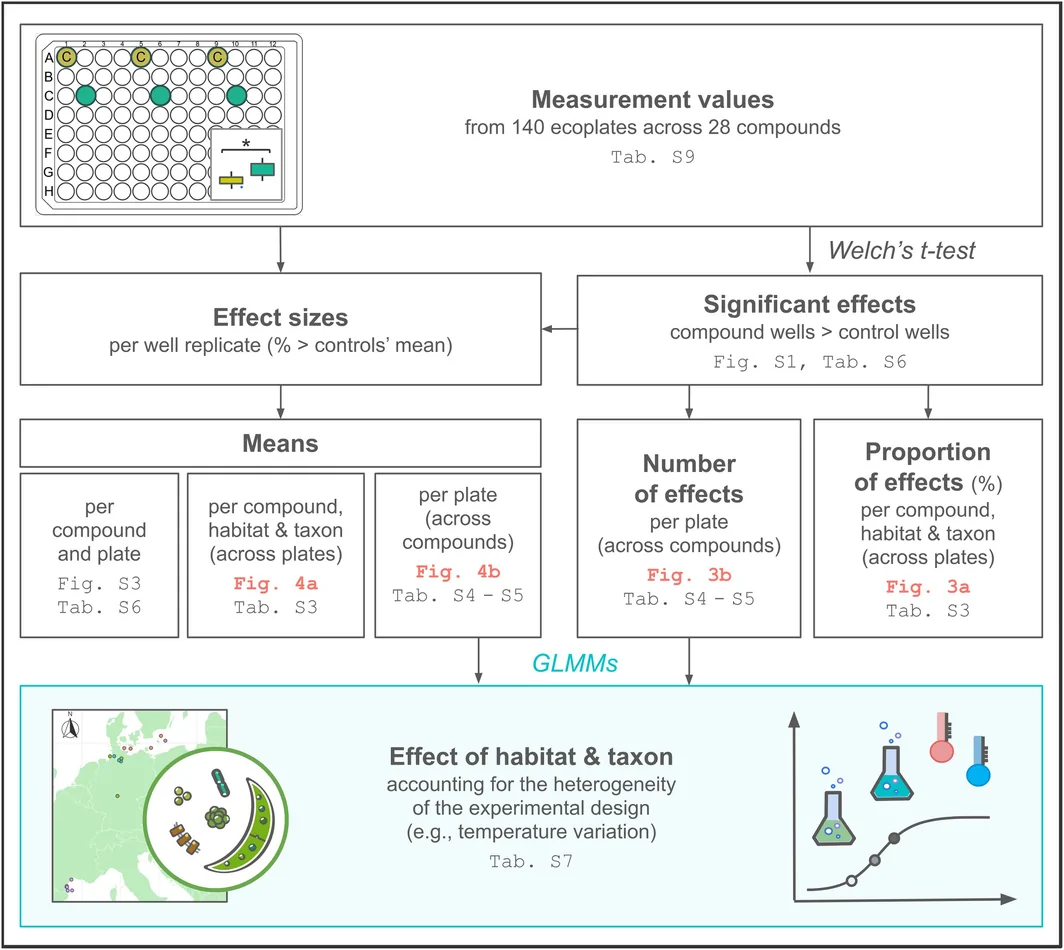
Statistical workflow. Figure and table references indicate where the data is presented. Significance (*) is defined by *p* ≤ 0.05. GLMMs = generalized linear mixed‐effects models. Full model specifications and test parameters are detailed in the main text as well as Table S7. The 96‐well plate graphic is public domain (CC0); map generated in R.

We used Biolog ecoplates to analyse the potential use of organic compounds. Ecoplates are 96‐well plates containing 31 different dissolved organic compounds covering different compound groups (e.g., carboxylic acids, amino acids) alongside wells without added organics serving as controls, each in triplicate (Table S2, Figure 2). Plates were inoculated with phytoplankton culture and after 24 h incubation, culture density was measured with different techniques (cytometry, photometry; see Figure 1b, Tables S1 and S9). Differences in the measurement results of wells containing organic compounds compared to those exclusively containing the phytoplankton culture (i.e., controls) were finally interpreted as growth responses of phytoplankton to the respective substances (see detailed explanation in Martens et al. 2024). Notably, in the present study, two adjustments were made to the standard ecoplate setup: (1) three compounds were omitted as their optical properties may interfere with the photometric measurements, resulting in a total of 28 compounds included (see Table S2); and (2) in the Baltic Sea and bog datasets, only two of the wells without organics were used as controls.

All strains were kept in nutrient enriched media that contained vitamins but no further organic substances and kept in a 12 h:12 h light: dark cycle with approximately 70–150 μmol photons s^−1^ m^−2^ in the light phase (Table S1). Strains were non‐axenic as we know that some of them need their microbiome to survive (Pang et al. 2022; Yao et al. 2019), or reacted sensitively to antibiotics, for example *Ostreococcus* (from previous unpublished data) (see also Section 4.4). Detailed information about the laboratory procedures is also given elsewhere (Listmann et al. 2021; Martens et al. 2024).

### 2.3 Statistical Analysis

**FIGURE 3:**
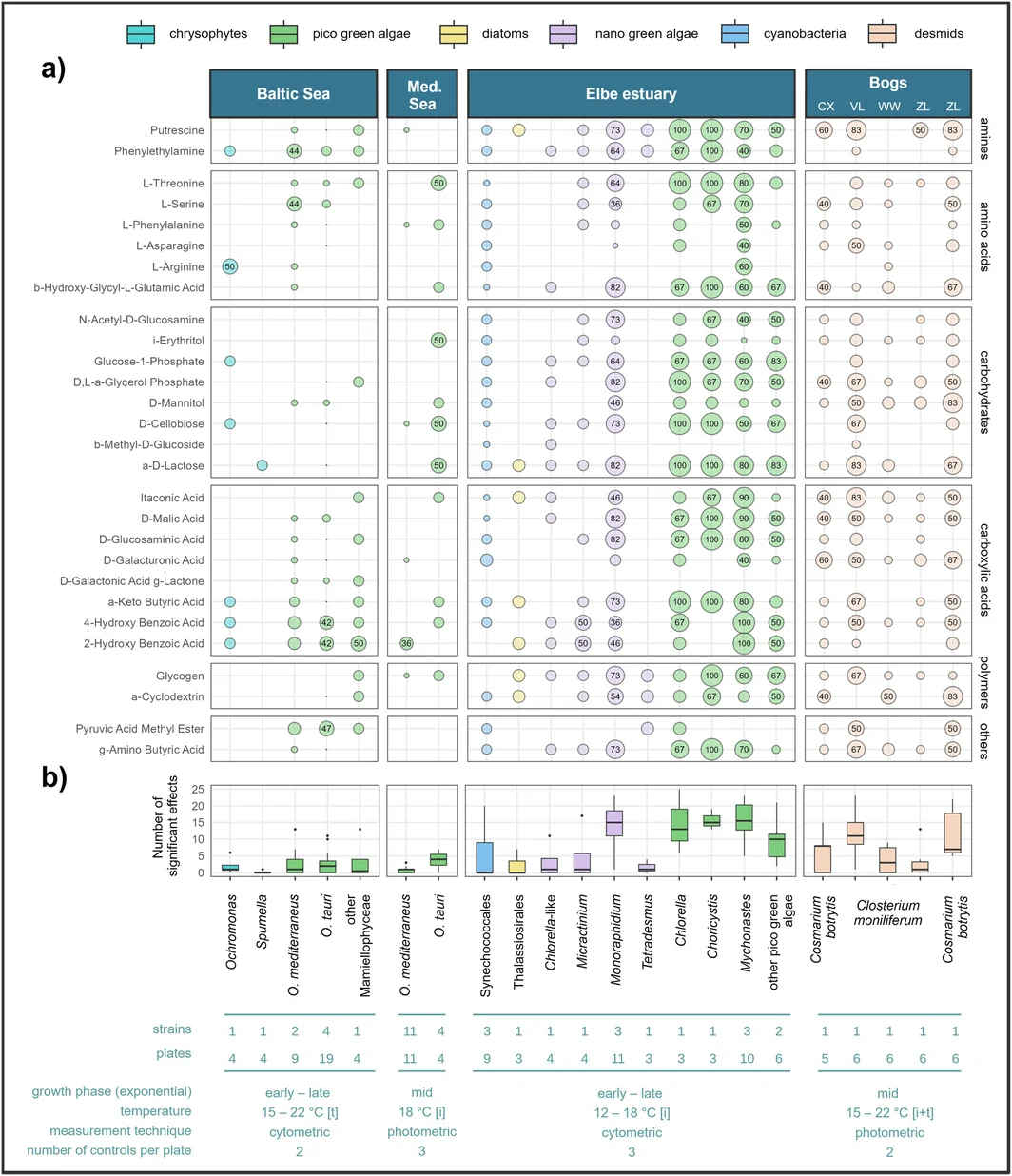
Observed effects in the presence of organic compounds. Points in (a) show the proportion of significant effects (one‐sided Welch's *t*‐test, *p* ≤ 0.05, *n* = 3 for compounds, *n* = 2–3 for controls) across the plates of each phytoplankton group. Numerical values are shown where > 33.3%. In (b), we show the total number of significant effects per plate. At the bottom, we indicate the number of plates and strains included as well as methodological aspects (Figure 1b). [*i*] = measurements at the strains' incubation temperatures, [*t*] = temperature treatments. CX = Cuxhaven, VL = Vlasina Lake, WW = Wohldorfer Wald and ZL = Zeller Loch. Details see Supporting Information (Figures S1, S2, and S4–56, Tables S1–S6).

**FIGURE 4:**
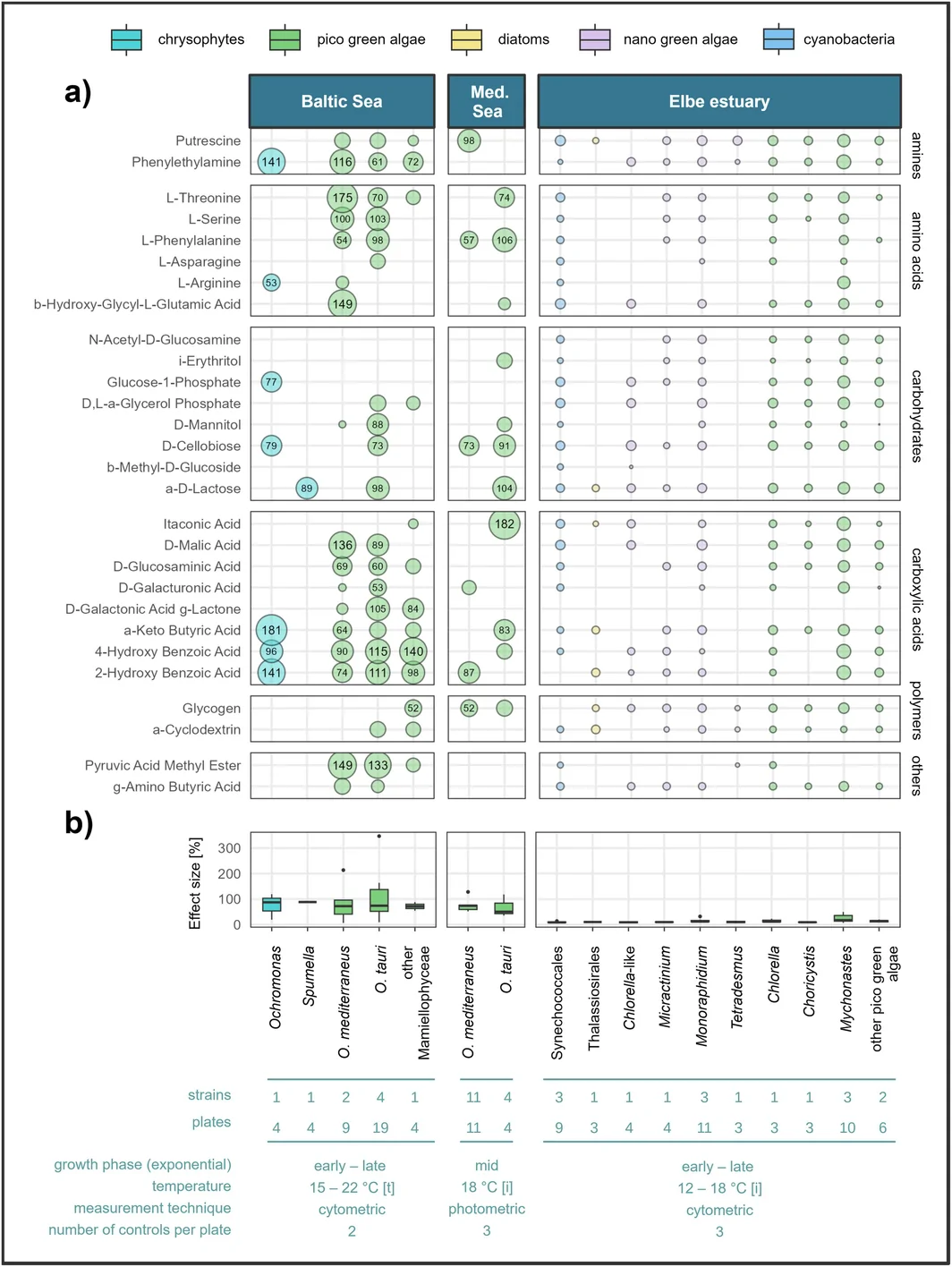
Observed effect sizes in the presence of organic compounds. Points in (a) show the average effect sizes of significant effects (one‐sided Welch's *t*‐test, *p* ≤ 0.05, *n* = 3 for compounds, *n* = 2–3 for controls) across the plates of each phytoplankton group. Numerical values are shown were > 50%. Standard deviations are shown in Table S3. In (b) we show the average effect sizes per plate. At the bottom, we indicate the number of plates and strains included as well as methodological aspects (Figure 1b). [*i*] = measurements at the strains' incubation temperatures, [*t*] = temperature treatments. Effect size was not obtained for the bogs. Details see Supporting Information (Figures S3–S6, Tables S1–S6).

Data analysis was carried out in R (version 4.4.1). We applied approximately 4000 one‐sided Welch's *t*‐tests (t.test() from stats version 4.4.1) with *p* ≤ 0.05 to analyse if measurement values (e.g., cell counts; see Figure 1b, Table S1) on the plates were higher in the wells with different organic compounds (*n* = 3 per comparison) than in the controls (*n* = 2–3 per comparison depending on the dataset; see Section 2.2, Figure 1b) (Figure 2). We consider these significantly positive effects indicative of the use of the respective compounds. Welch's method was specifically chosen over standard *t*‐tests because it does not assume equal variances between groups. For further analyses, we counted the number of significant effects observed on each ecoplate (Figure 2). The results are shown in Figure 3b, grouped by taxon and habitat (see also Tables S4 and S5). In Figure 3a we present the proportions of significant effects per compound, habitat and taxon, across plates (%) (see also Table S3, Figure 2). Moreover, we provide information about the effect sizes of the significant effects. Effect size is expressed as % above the controls' mean, and generally presented as means (Figure 2). Figure 4b shows the mean effect sizes per plate, grouped by taxon and habitat (see also Tables S4 and S5). Figure 4a shows the average effect sizes per compound, habitat and taxon, across plates (see also Table S3). In the bog data, control values were partially negative, which did not allow us to obtain effect sizes.

The 28 compounds covered various compound groups (e.g., carbohydrates, amino acids) and, more broadly, P, N and sole C sources, with varying numbers of compounds each (Table S2). Pearson correlation analysis (package ggpubr, version 0.6.0) revealed that the number of significant effects per compound group and elemental source generally correlated with the total number of effects per plate (Figures S7 and S8). Therefore, further group‐specific statistical tests were omitted.

We used generalized linear mixed‐effects models (GLMMs; detailed in Table S7) to analyse how habitat origin and taxonomic affiliation affected the number of significant effects per plate (GLMM_1, GLMM_3; data from all 140 plates) and the average effect size per plate (GLMM_2, GLMM_4; data from 78 plates), while accounting for experimental heterogeneity (Figure 2). This heterogeneity was driven by uneven coverage of temperatures and growth phases, baseline biological variation among strains, and—in the habitat models (GLMM_1 and GLMM_2)—differences in taxonomic representation per habitat. Temperature and growth phase were included as random effects throughout all models. However, because some datasets were restricted to specific conditions, these two variables were combined into a single random effect (Table S7). In the habitat models (GLMM_1 and GLMM_2), habitat was the sole fixed effect. For GLMM_1 (response: number of effects), strain nested within taxon was included as an additional random effect to account for taxa selection across habitats. To prevent model singularity, this random effect was omitted from GLMM_2 (response: effect size), which contained fewer observations because effect sizes were only calculated for plates showing significant effects and generally unavailable for the bog dataset. In the taxa models (GLMM_3 and GLMM_4), the interaction between taxon and habitat was set as the fixed effect. This allowed us to determine whether different taxa from the same habitat responded differently, and whether the behaviour of taxa represented across multiple habitats (*O. tauri*, *O. mediterraneus* , *C. botrytis* and *C. moniliferum* ) varied by origin. To account for baseline biological variation, strain was included as an additional random effect in GLMM_3 (response: number of effects). As with the habitat models, this random effect was excluded from GLMM_4 (response: effect size) due to lower sample sizes and resulting convergence issues.

All models were fitted using the glmer() function from the lme4 package (version 1.1‐35.5). A Poisson distribution was used to model the number of significant effects per plate, while a Gamma distribution was used for effect sizes (%); both employed a log link function. To ensure convergence, models were run using the BOBYQA optimizer with a maximum of 100,000 iterations. For all models, significance of fixed effects was assessed using Type III Wald chi‐square tests (Anova() from the package car, version 3.1‐3), while overall model fit was evaluated using the marginal and conditional *R* ^2^ values (r2() from the package performance, version 0.17.1). Post hoc pairwise comparisons and estimated marginal means were analysed using emmeans() from the emmeans package (version 2.0.3), with *p*‐values adjusted for multiple comparisons using the Tukey method and 95% confidence intervals calculated for all reported estimates. Complete statistical outputs for all models and post hoc tests are provided in Table S7.

Figures were produced with ggplot2 (3.5.1), svglite (version 2.1.3), patchwork (version 1.3.2.), rnaturalearth (version 1.1.0), sf (version 1.0–21) and ggspatial (version 1.1.9). Data preparation was carried out using tidyverse (version 2.0.0). We also used LibreOffice Draw (version 7.1.2.2) and Google Slides for overview figures and the addition of text notes as well as Gemini 3.1 Pro (Google) and ChatGPT (GPT‐5) to streamline R code and to check the finalized manuscript for common grammatical and typographical errors.

## 3 Results

### 3.1 Observed Effects Across Taxa and Habitats

In 41 of 46 strains (89%), at least one compound on at least one ecoplate was associated with significantly higher measurement values than the control (*p* ≤ 0.05) (Figure 3a,b, Tables S3–S6, Figures S1 and S2). Effect sizes varied from 3% to 347% (Figure 4a,b, Table S6, Figure S3). Five *Ostreococcus* strains from the Mediterranean Sea did not exhibit significant effects in the presence of compounds (Figure S2, Table S5). The highest number of significant effects on a single plate was 25, observed for *Chlorella* from the Elbe estuary (Figure 3b, Table S4). Effects were found for all compound groups, as well as for N, P and sole C sources (Figure 3a, Tables S2 and S6). Effects appeared for every single compound, often across a wide range of taxa and habitats (Figure 3a). For instance, 4‐Hydroxy Benzoic Acid was associated with significant effects in 17 of the 22 phytoplankton groups shown in Figure 3a, covering all included functional groups and habitats. For some compounds, significant effects rarely appeared across habitats, especially for b‐Methyl‐d‐Glucoside, d‐Galactonic Acid g‐Lactone and l‐Arginine (Figure 3a).

### 3.2 Habitat‐ and Taxon‐Specific Results

The habitat GLMMs (GLMM_1 and GLMM_2) show that—despite the heterogeneity imposed by different experimental designs—habitat was a significant factor, accounting for 44% of the variation in the number of significant effects per plate and 67% of the variation in average effect sizes according to the marginal *R* ^2^ (*p* ≤ 0.05 for both; for details, see Table S7). Post hoc pairwise comparisons of model‐adjusted means revealed that the Elbe estuary exhibited a significantly greater number of effects than both marine habitats (GLMM_1, *p* ≤ 0.05), but significantly smaller effect sizes per plate (GLMM_2, *p* ≤ 0.05) (Table S7; see also Figure S4). All other habitat comparisons were non‐significant, with the exception of the number of effects being overall higher in the bog near Vlasina Lake compared to the Mediterranean Sea (GLMM_1, *p* ≤ 0.05; Table S7, Figure S4). Species isolated from different habitats sometimes behaved differently with respect to the number of effects per plate (GLMM_3, *p* ≤ 0.05; Table S7). For instance, *O. mediterraneus* from the Mediterranean Sea exhibited fewer effects than *O. mediterraneus* from the Baltic Sea (Table S7, see also Figure 3b). Similarly, *C. moniliferum* from the bog near Vlasina Lake had more effects than the *C. moniliferum* strain isolated from Zeller Loch (Table S7, Figure 3b).

While taxonomic affiliation generally did not significantly influence effect size in the post hoc comparisons—except when comparing *Mychonastes* and Synechococcales from the Elbe estuary (GLMM_4, Table S7; see also Figure 4b, Table S4)—various taxa within the same habitat differed significantly in their total number of effects (GLMM_3, *p* ≤ 0.05, Table S7). For instance, in the Elbe dataset, *Mychonastes* and *Monoraphidium* had significantly more effects per plate than *Tetradesmus* and the Thalassiosirales strain (see also Figure 3b). In other habitats, differences were observed between *C. moniliferum* and *C. botrytis* (bog Zeller Loch), as well as between *O. mediterraneus* and *O. tauri* (Mediterranean Sea) (Table S7, Figure 3b).

The higher number of effects achieved in certain phytoplankton groups might be explained by two different factors: (1) effects appeared across a broad range of compounds (number of points per taxon and habitat in Figure 3a); and (2) results were consistent across plates (point size in Figure 3a). For instance, *Mychonastes* experienced significant effects in the presence of 25 different compounds (out of 28 compounds included), and 2‐Hydroxy Benzoic Acid was associated with significant effects on all 10 plates (i.e., 100%) covering different strains, growth phases and bioreplicates (Figure 3a, Figure S2). In contrast, various other taxa across habitats—including all marine taxa—yielded less consistent results across plates and covered a smaller number of different compounds (Figure 3a). *Ostreococcus mediterraneus* from the Mediterranean Sea, for example, experienced significant effects in the presence of only six different compounds. Inconsistent results generally stem from varying behaviour across different strains, temperatures, growth phases and bioreplicates (Table S1). For instance, Synechococcales from the Elbe estuary exhibited more effects in the mid exponential phase with strain C6 contributing more effects than the other two strains while Thalassiosirales exclusively showed effects in the late exponential phase (Figure S5). Results from different bioreplicates of the same strain and growth phase also sometimes differed notably, for example, 5 versus 21 effects on the two late phase plates of *Mychonastes* strain P6 (Figure S2). Inconsistencies observed among the bog desmids resulted from variability across temperatures and bioreplicates (Figure S6). In the Baltic Sea dataset, low consistency was linked to temperature and growth phase. Specifically, significant effects were observed less frequently at higher temperatures and later growth phases (Figures S5 and S6). Furthermore, the two strains of *O. mediterraneus* behaved quite differently from one another, with strain 19.5 yielding almost no effects, and strain 4.3 yielding among the highest number of effects across the Baltic Sea strains (Figure S2). We also observed high strain variability among the *O. tauri* strains from the Mediterranean Sea (Figure S2).

### 3.3 Compound‐Specific Analysis

Although the number of effects observed per compound group (e.g., carboxylic acids) and source type (i.e., C, N, or P) generally correlated with the total number of effects per plate (Pearson correlation, *p* ≤ 0.05; Figures S7 and S8), we observed a few distinct deviations from this overall trend.

Baltic Sea strains exhibited fewer effects in the presence of carbohydrates and disproportionately more with carboxylic acids (Figure 3a, Figure S8). Even on plates with a high total number of effects, carbohydrate‐driven effects remained consistently low (slope = 0.1; Figure S8). Conversely, the number of effects driven by carboxylic acids steeply increased along the number of total effects (slope = 0.9; Figure S8). This pattern was specific to the Baltic Sea. In the Mediterranean Sea, it was rather reversed. However, results have to be interpreted carefully, as Mediterranean Sea strains never reached more than seven effects in total, and included a lower number of plates (15) (Figure S8, Table S4).

While N as well as sole C sources were overall often associated with significant effects across habitats (Figure 3a, Table S2)—correlating with the total number of effects per plate (Figure S7)—this was not always the case for the 2 P sources (Glucose‐1‐Phosphate, d,l‐a‐Glycerol Phosphate). Unlike in the freshwater habitats, in the marine strains the number of effects associated with the P sources was rather meagre (Figure 3a, Figure S7). For the Baltic Sea strains, this is in line with the lower number of effects caused by carbohydrates, the compound group both P sources belong to. However, in the Mediterranean Sea, various carbohydrates were repeatedly associated with significant effects, yet neither of the 2 P sources was associated with any (Figure 3a, Figure S7).

Ultimately, some compound‐specific results showed notable differences between the two marine habitats. For instance, Phenylethylamine, l‐Serine and Pyruvic Acid Methyl Ester were associated with significant effects across several plates of Baltic Sea *Ostreococcus*—spanning different growth phases and temperature treatments—but this was never observed for the Mediterranean Sea. The results for Pyruvic Acid Methyl Ester are particularly distinct. While large effect sizes were observed across the Baltic Sea plates (Figure 4a), the vast majority of measurements for the Mediterranean Sea remained below control levels (Table S9).

## 4 Discussion

### 4.1 Osmomixotrophy as a Ubiquitous Trait in Phytoplankton

**FIGURE 5:**
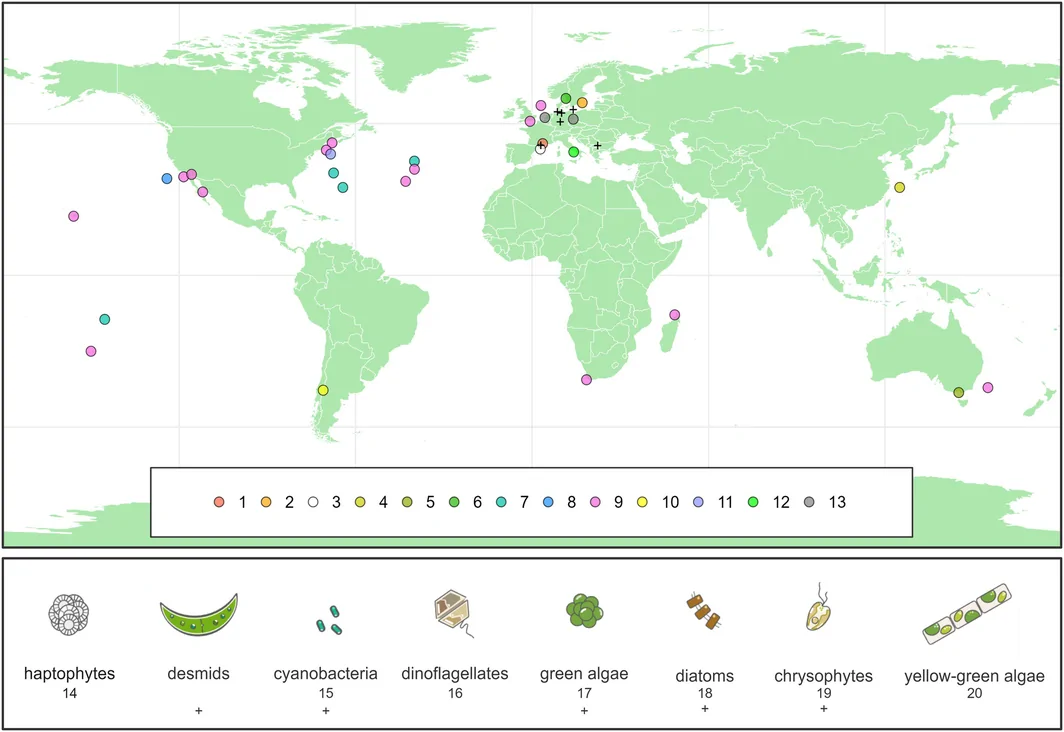
Reported osmomixotrophic behaviour (non‐exhaustive). Data points on the map show the approximate areas from which phytoplankton with reported osmomixotrophic behaviour originated. Below, we show phytoplankton groups with reported osmomixotrophic behaviour. Data from this study (+) and previous research: 1 = Foresi et al. (2022), 2 = Hagström et al. (2021), 3 = Mena et al. (2024), 4 = Zhang et al. (2021), 5 = Liu et al. (2024), 6 = Villanova et al. (2024), 7 = Muñoz‐Marín et al. (2020), 8 = Palenik et al. (2006), 9 = Godrijan et al. (2020), 10 = Beamud et al. (2014), 11 = Balch et al. (2023), 12 = Meyer et al. (2022), 13 = Spijkerman et al. (2017), 14 = Balch et al. (2023); Beamud et al. (2014); Godrijan et al. (2020), 15 = Hagström et al. (2021); Muñoz‐Marín et al. (2020); Palenik et al. (2006), 16 = Li et al. (2021); Mena et al. (2024); Zhang et al. (2021), 17 = Abdullin and Bagmet (2015); Beamud et al. (2014); Foresi et al. (2022); Hagström et al. (2021); Patnaik and Mallick (2020); Spijkerman et al. (2017), 18 = Abdullin and Bagmet (2015); Mena et al. (2024); Meyer et al. (2022); Villanova et al. (2024); Zhu et al. (2024), 19 = Hiltunen et al. (2012), 20 = Liu et al. (2024). See also Table S8. Map generated in R. A comprehensive map of genes potentially involved in organic matter transport in *Synechococcus* has been provided elsewhere (Muñoz‐Marín et al. 2024).

Osmomixotrophic behaviour has been reported for different phytoplankton groups from various locations around the world (Figure 5, Table S8). Previous studies have provided genetic evidence for phytoplankton possessing the physiological toolkits to use organic compounds (Hagström et al. 2021; Li et al. 2021; Muñoz‐Marín et al. 2024; Palenik et al. 2006; Zhang et al. 2021; Zhu et al. 2024). Some studies integrate DOM acquisition into the bigger picture of metabolic processes (Foresi et al. 2024; Villanova and Spetea 2021). The results of the current synthesis, including growth response data from 46 phytoplankton strains across taxa, functional groups and ecosystems contribute further indication of ubiquitous organic matter acquisition. Unlike proposed in the past, the uptake of organic compounds is not limited to certain essential or limiting substances, such as vitamins, or N and P sources. The observations indicate that osmomixotrophy is not strictly linked to certain habitats, functional groups or taxa, but rather universal.

Previous studies have shown that osmomixotrophic processes in phytoplankton involve genes commonly found in heterotrophs (e.g., animals, bacteria, fungi), for instance genes encoding membrane transporters such as the urea transporter DUR3 (Li et al. 2021) or the amino acid transporter families SLC7, SLC36, SLC38 and AAPJ (Li et al. 2021; Zhang et al. 2021). Altogether, the universal appearance of osmomixotrophy and the presence of orthologous genes across domains and lineages may imply that heterotrophic processes in phytoplankton represent retained ancestral traits, rather than something that certain phytoplankton taxa acquired over time. Previous studies have shown that many eukaryotic genes are deeply conserved across very distantly related lineages, so that even seemingly unrelated organisms, such as phytoplankton and animals, share homologous genes inherited from their common eukaryotic ancestor (see e.g., Armbrust et al. 2004; Merchant et al. 2007). However, current genetic support for a link between osmomixotrophic traits and heterotrophic ancestry is limited to a few involved transporters rather than a broader suite of metabolic pathways. Indeed, some of these traits may result from novel genes that emerged as adaptations to specific environmental pressures, or through other mechanisms such as horizontal gene transfer (see e.g., Bowler et al. 2008; Milner et al. 2019).

### 4.2 Acquisition of Organic Compounds Is Not Strictly Tied to Low Nutrient or Light Availability

Mixotrophic behaviour has been proposed as a strategy to deal with photosynthetically inconvenient conditions such as nutrient deficiencies or light limitation (Calderini et al. 2022; Foresi et al. 2022; Li et al. 2021; Tuchman et al. 2006). Recent studies show that the expression of membrane transporters for the uptake of organic compounds can be upregulated during N or CO_2_ deficiencies (Li et al. 2021; Zhang et al. 2021). The results of the present study indicate that nutrient and light limitation—conditions not imposed by the experimental design—are not prerequisites for osmomixotrophy. For some of the Elbe estuary strains, we previously showed that the use of compounds was not increased under light reduction (Martens et al. 2024). Furthermore, the widespread utilization of sole C sources indicates that nutrient scavenging was not the exclusive driver for compound acquisition. Extensive C‐source utilization has also been documented for various coccolithophore strains from different marine habitats (Godrijan et al. 2020).

### 4.3 Habitat and Taxonomic Affiliation May Explain Certain Osmomixotrophic Traits

Our results suggest that phytoplankton's osmomixotrophic abilities are associated with their taxonomic affiliation and habitat of origin. This might be explained by the strains' evolutionary history, depending on the selection pressures their ancestors faced in both previous and current environments. Such selection pressures may include the availability of compounds—such as those derived from terrestrial runoff—their utility in metabolic cycles, the associated metabolic costs and how they align with other environmental factors, such as light and nutrient availability. Generally, mixotrophic functions may be expanded through mechanisms such as gene duplication events, the emergence of novel genes, or horizontal gene transfer, or lost as an evolutionary trade‐off to optimize other crucial phenotypic traits.

Mechanistically, an adaptation to steady DOM supply (Hopple et al. 2019; Novak et al. 2019; Roth et al. 2014; Tobias‐Hünefeldt et al. 2024; Watras and Hanson 2023)—and likely higher complexity of DOM due to strong terrestrial links (Kellerman et al. 2014; Roth et al. 2014)—might explain the ability of many strains from the Elbe estuary and the bogs to utilize a broad range of compounds, across growth phases and temperatures. In that sense, their acquisition of organic compounds appears largely constitutive. By contrast, Baltic Sea strains used compounds more often under specific conditions, precisely in the early exponential phase and at lower temperatures, which we previously attributed to low photosynthetic efficiency (Listmann et al. 2021). Their osmomixotrophic behaviour appears compensatory. The higher effect sizes found in the marine strains support this theory: Where DOM is less available (Amaral et al. 2023; Hoikkala et al. 2015; Lodeiro et al. 2021; Seidel et al. 2017)—but needed for compensatory demands—a rapid response is necessary to remain competitive. Nevertheless, drawing ultimate conclusions about mechanistic relationships remains challenging without further research. Differences between the habitats—even when statistically significant—should be interpreted with caution, given the small number of included taxa—especially from the marine habitats—and the distinct ecological niches these strains may occupy. Previous studies show that other marine phytoplankton such as coccolithophores are able to utilize a broad range of organic compounds (Godrijan et al. 2020).

The similarities in behaviour observed across strains from the same taxonomic group (e.g., *Mychonastes*, *Ostreococcus*) may be explained by ancestry, but could also result from independent adaptations driven by similar habitat history or shared baseline physiology. For instance, the meagre—or possibly more specialized—utilization of organic compounds in *Ostreococcus* may be an artefact of their inherited small genome size (Derelle et al. 2006). The small genome may help them efficiently manage genomic resource demands (e.g., for P), but might come with the trade‐off of encoding fewer metabolic pathways. However, the limited divergence between the Baltic Sea and Mediterranean Sea strains could reflect similar habitat conditions: Both are semi‐enclosed marine habitats with similar DOM concentrations in the relevant areas (e.g., western Baltic Sea, Mediterranean lagoons; see also Table S1) (Amaral et al. 2023; Hoikkala et al. 2015; Lodeiro et al. 2021; Seidel et al. 2017).

Nevertheless, specific differences found between taxa isolated from different habitats—for example, the exclusive use of Pyruvic Acid Methyl Ester by Baltic Sea *Ostreococcus* strains supports habitat‐specific traits. Yet, without further physiological evidence and a deeper understanding of present habitats, for example, with respect to DOM composition, the underlying mechanisms remain speculative. Similarly, we lack further relevant data about the specific bog habitats our desmids originated from, which could help explain observed differences, such as the extensive osmomixotrophic abilities of the Serbian strain of *C. moniliferum* in contrast to both German strains.

### 4.4 Constraints of the Study Design

In the present analysis, we summarize data from different previously published studies and newly acquired datasets. These originated from multiple research projects, with varying objectives, leading to strong heterogeneity in the experimental design (see also Figure 1b). While the applied analyses show that our results are statistically robust, they cannot make up for experimental shortcomings. For instance, while the GLMMs can integrate taxonomic variations from the different habitats, they cannot compensate for the fact that the marine habitats and the bogs were each represented by two main, closely related taxonomic groups, and that some taxa in our study were represented by single strains. We simply do not know how other taxa and strains would behave. Integrating further phyla—including those from other functional groups such as diatoms—as well as additional strains for taxa currently represented by only one strain (e.g., *Tetradesmus*, *Ochromonas*), could alter the observed patterns and conclusions. Similarly, given the differential behaviour observed across taxa and habitats in response to experimental variables such as growth phase and temperature, it remains unclear how strains would respond to untested conditions. For instance, it remains challenging to fully anticipate how *Ostreococcus* from the Mediterranean Sea would respond to a different temperature, or how the bog desmids might behave in a different growth phase.

Another potential limitation relates to the statistical assessment of compound utilization via Welch's *t*‐tests. With approximately 4000 individual comparisons, a certain proportion of statistically significant results likely occurred by pure chance (false positives). However, corrections for multiple comparisons (e.g., the Benjamini‐Hochberg procedure) could not be applied uniformly due to the varying number of controls (*n* = 2–3). Because datasets with *n* = 2 (the bogs and Baltic Sea) have inherently lower degrees of freedom, the lowest achievable *p*‐values are inherently restricted. Therefore, applying a stringent false discovery rate penalty across the data would disproportionately penalize and entirely erase the effects observed in the *n* = 2 habitats, masking true biological signals purely due to sample size constraints. Furthermore, data could not be pooled prior to the *t*‐tests (e.g., across plates) to artificially increase sample sizes, as biological behaviour varied considerably between plates for several taxa. Consequently, individual significant data points—especially those appearing on a single plate for a specific taxon and habitat (indicated by small point sizes in Figure 3a)—should be interpreted with caution and not treated as conclusive evidence of compound utilization. In contrast, significant effects that were consistent across multiple plates, such as those observed for *Mychonastes* from the Elbe estuary or *C. moniliferum* from near Vlasina Lake, can be considered reliable. Finally, while taxa with only two controls had mathematically lower statistical power to generate significant results in the initial *t*‐tests, there was no systematic bias tying the overarching patterns to the number of controls: low numbers of effects were recorded in both the Mediterranean Sea (three controls) and the Baltic Sea (two controls), while high numbers were observed in both the Elbe estuary (three controls) and the bogs (two controls).

Just as some significant effects may arise by chance, a lack of statistical significance in the *t*‐tests does not definitively prove that strains were unable to utilize specific organic compounds. Instead, non‐significance can result from high variation across replicate wells. With small sample sizes (*n* = 3), strong stochastic variation within a single well can inflate the overall variance, masking true biological effects even when individual wells exhibit measurements clearly above the controls' mean. This variability differed notably among datasets; for example, the average standard deviation across replicates was approximately 25% in the marine habitats, compared to only 5% in the Elbe estuary. Also, as described in our previous work (Martens et al. 2024), microbial toxin production in the non‐axenic cultures may have inhibited phytoplankton growth in the presence of certain organic compounds, potentially masking their positive effects. These factors may have led to false negatives. One example could be the previously reported ability of *O. tauri* from the Mediterranean Sea to utilize l‐Arginine (Foresi et al. 2022), which was not reflected in our results.

Further methodological heterogeneity arises from the use of different measurement techniques (Figure 1b). Importantly, the results do not show a distinct bias where one method consistently yielded stronger or more frequent effects. High numbers of significant effects were detected using both flow cytometry for the Elbe estuary and photometry for the bogs. Similarly, Baltic Sea strains (measured via cytometry) and Mediterranean Sea strains (measured via photometry) both yielded high effect sizes, but a low overall number of effects. Nevertheless, we cannot completely rule out more subtle biases. While both techniques act as proxies for population growth, direct cell counts (cytometry) and pigment‐based measurements (photometry) do not always align. For instance, osmotrophic resource acquisition is known to induce physiological adaptations, including alterations in cellular chlorophyll content and cell size (Azaman et al. 2017). Such natural phenotypic shifts can affect the comparability of cytometric and photometric approaches.

The design of the ecoplates as such should also be taken into consideration. Although these plates screen a wide range of substances, natural DOM in aquatic habitats is vastly more complex. Consequently, a strain's response to the specific compounds provided may not fully capture its broader osmomixotrophic potential in real ecosystems. Findings related to compound and source groups represented by only two compounds on the plates—such as amines and P sources—must therefore be interpreted with particular caution. For instance, the apparent low affinity of marine taxa for the P sources on the ecoplates—also reported in previous research (Godrijan et al. 2020)—may reflect the inability to utilize those two exact substances (Glucose‐1‐Phosphate, d,l‐a‐Glycerol Phosphate). We should not conclude that oceanic strains generally refuse P sources, especially when P is a limiting factor—a condition not imposed by the study design.

Finally, study limitations result from the inclusion of non‐axenic strains. While these cultures more closely reflect natural ecological conditions, they limit our ability to definitively interpret how phytoplankton utilized the provided compounds (e.g., direct use vs. use of metabolites from microbial breakdown). Although expected patterns for microbial NH_4_ and CO_2_ supply—such as more effects in the presence of N sources and towards the late exponential phase—were absent, and ampicillin treatments did not alter the number of observed effects in previous trials (Figure S9; for details, see (Martens et al. 2024)), microbial effects cannot be fully excluded. Ultimately, disentangling different factors—for example, growth inhibition by microbial toxins, growth promotion by bacterial‐derived metabolites and actual direct use of the provided compounds—requires methodological approaches not applied in the present study. Notably, previous work with axenic cultures has already demonstrated microbiome‐independent compound uptake in taxa directly relevant to our study, such as *Ostreococcus* (Foresi et al. 2022, 2024), *Mychonastes* (Abdullin and Bagmet 2015) and *Tetradesmus* (Patnaik and Mallick 2020). Furthermore, broader studies employing transcriptomics, isotopic labelling and axenic growth assays have confirmed the direct utilization of compounds across various other phytoplankton taxa (Hagström et al. 2021; Li et al. 2021; Liu et al. 2024; Mena et al. 2024; Palenik et al. 2006; Zhang et al. 2021; Zhu et al. 2024).

## 5 Conclusion

Understanding phytoplankton's metabolic processes and environmental drivers is fundamental to determine their functions in ecosystems and accurately assess their role in carbon cycling and climate‐related ecosystem processes. The present synthesis highlights the prevalence of osmomixotrophy in terms of dissolved organic compounds in phytoplankton across taxa and habitats. Our results suggest that future experimental design in phytoplankton research must move beyond the strict autotrophic paradigm to account for carbon flux from dissolved organic sources. With DOM being one of the largest carbon pools on Earth (Hansell et al. 2009; Houghton 2003) and phytoplankton being key mediators of carbon sequestration (Basu and Mackey 2018), this study underscores the ecological significance of heterogeneous nutritional strategies and the necessity to address existing knowledge gaps to finally draw conclusions about the impact of osmomixotrophy in the context of single ecosystems and on global scales.

## Author Contributions

**Nele Martens:** conceptualization, methodology, software, writing – original draft, writing – review and editing, formal analysis, data curation, investigation, visualization. **Luisa Listmann:** conceptualization, methodology, writing – review and editing, data curation, investigation, validation. **Jette Ludewigs:** investigation, writing – review and editing, validation. **C.‐Elisa Schaum:** writing – review and editing, funding acquisition, validation, resources, conceptualization.

## Funding

This work was supported by Deutsche Forschungsgemeinschaft (407270017/RTG2530) and Open Access Publication Fund of Universität Hamburg.

## Conflicts of Interest

The authors declare no conflicts of interest.

## Supporting information


**Figure S1:** Significant effects (green) of each compound on each ecoplate. Significant effects (*p* ≤ 0.05) were determined using one‐sided Welch's *t*‐tests with 3 replicates per compound and 2–3 replicates per control. The horizontal scale shows the different compounds (bottom) sorted by compound group (top). On the vertical axis we show the different plates which include different taxa, strains, incubation times, temperatures, growth phases and replicates. Precisely, it is named as such: >>taxon—strain—temperature—growth phase (1–3 = early—late)—replicate<<. The vertical panels show different ecosystems (BS = Baltic Sea, Bo = Bogs, EE = Elbe estuary, MS = Mediterranean Sea) and functional groups (Chry = chrysophytes, Cya = cyanobacteria, Des = desmids, Dia = diatoms, NG = nano green algae, PG = pico green algae) (see also Table S1). For the bog desmids, different origin is indicated (CX = Cuxhaven, VL = Puddly near Vlasina Lake, WW = Wohldorfer Wald, ZL = Zeller Loch).
**Figure S2:** Number of significant effects per ecoplate clustered by strains covering different taxa from different habitats. Significant effects (*p* ≤ 0.05) were determined using one‐sided Welch's *t*‐tests with 3 replicates per compound and 2–3 replicates per control. Colour scheme of the boxplots indicates functional group (Chry = chrysophytes, Cya = cyanobacteria, Des = desmids, Dia = diatoms, NG = nano green algae, PG = pico green algae). Colour scheme of the points indicates the experimental temperature. Point shape shows the timepoint during the exponential phase. Note that effect size was not obtained for the bog dataset, explaining the absence of ‘+’ here. For the bog desmids, different origin is indicated (CX = Cuxhaven, VL = Puddly near Vlasina Lake, WW = Wohldorfer Wald, ZL = Zeller Loch).
**Figure S3:** Effect sizes of each compound with significant effects per ecoplate (means of well triplicates; SD see Table S6). Significant effects (*p* ≤ 0.05) were determined using one‐sided Welch's *t*‐tests with 3 replicates per compound and 2–3 replicates per control. Effect size shows how much higher the measurement value (cell count, fluorescence) was in the presence of organic compounds compared to the control [%]. The horizontal scale shows the different compounds (bottom) sorted by compound group (top). On the vertical axis we show the different plates which include different taxa, strains, incubation times, temperatures, growth phases and replicates. Precisely, it is named as such: >>taxon—strain—temperature—growth phase (1–3 = early—late)—replicate<<. The vertical panels show different ecosystems (BS = Baltic Sea, EE = Elbe estuary, MS = Mediterranean Sea) and functional groups (Chry = chrysophytes, Cya = cyanobacteria, Dia = diatoms, NG = nano green algae, PG = pico green algae) (see also Table S1). Effect size was not obtained for the bog dataset.
**Figure S4:** Number of significant effects per plate and average effect size per plate clustered by habitat. We here show the data across all plates (*n* = 140) including different taxa, temperatures, growth phases per habitat (for details see also e.g., Table S5, Figures S1–S3). Significant effects (*p* ≤ 0.05) were determined using one‐sided Welch's *t*‐tests with 3 replicates per compound and 2–3 replicates per control. Effect size shows how much higher the measurement value (cell count, fluorescence) was in the presence of organic compounds compared to the control. CX = Cuxhaven, VL = Puddly near Vlasina Lake, WW = Wohldorfer Wald, ZL = Zeller Loch. Note that effect size was not obtained for the bog dataset.
**Figure S5:** Number of significant effects along growth phases (1 = early, 2 = mid, 3 = late exponential). Significant effects (*p* ≤ 0.05) were determined using one‐sided Welch's *t*‐tests with 3 replicates per compound and 2–3 replicates per control. Panels show the different taxa, strains (e.g., 16.3) and temperatures. Colour scheme indicates functional group (Chry = chrysophytes, Cya = cyanobacteria, Dia = diatoms, NG = nano green algae, PG = pico green algae). Data points are connected by a simple line as a visual aid. Point size shows the mean effect size of significant effects per plate [%] as indicated above (no significant effects = no point). Where more than one bioreplicate was included, line is drawn through the mean. Strain and temperature combinations that did not cover multiple growth phases were removed, which also concerns the complete bog and Mediterranean Sea data.
**Figure S6:** Number of significant effects along temperatures. Significant effects (*p* ≤ 0.05) were determined using one‐sided Welch's *t*‐tests with 3 replicates per compound and 2 replicates per control. Panels show the different taxa, strains (e.g., 14.12) and growth phases. Colours indicate the functional group (Chry = chrysophytes, Des = desmids, PG = pico green algae), and for the desmids also different bioreplicates. Data points are connected by a simple line as a visual aid. For the Baltic Sea data point size shows the mean effect size of significant effects per plate [%] as indicated above. For the bog data point size is fixed where effects appeared, as effect size was not obtained. In both datasets, no point indicates no significant effects (i.e., *y* = 0). Note that strain 274 has only 2 data points in one of the replicates (15°C and 22°C). Strain and growth phase combinations that did not cover multiple temperatures were removed, which also concerns the complete Elbe estuary and Mediterranean Sea data. For the bog desmids, different origin is indicated (CX = Cuxhaven, VL = Puddly near Vlasina Lake, WW = Wohldorfer Wald, ZL = Zeller Loch).
**Figure S7:** C, N and P sources with significant effects per plate, plotted against the total number of significant effects on each plate (across all 140 included plates; per habitat). Significant effects (*p* ≤ 0.05) were determined using one‐sided Welch's *t*‐tests with 3 replicates per compound and 2–3 replicates per control. Note that plates contained 2 P sources, 11 N sources and 15 sole C sources. Data points partially overlap. This is visible by darker appearance of the overlapping semi‐transparent points. Labels show Pearson correlation coefficients (R) and *p*‐values. Regression slopes are constrained by the number of compounds per group and should only be compared between equally sized groups. For clarity, the data from the four bogs is pooled together.
**Figure S8:** Compounds belonging to the different compound groups with significant effects per plate, plotted against the total number of significant effects on each plate (across all 140 included plates; per habitat). Significant effects (*p* ≤ 0.05) were determined using one‐sided Welch's *t*‐tests with 3 replicates per compound and 2–3 replicates per control. Note that plates contained 2 amines, 6 amino acids, 8 carbohydrates, 8 carboxylic acids, 2 polymers and 2 other compounds. Data points partially overlap. This is visible by darker appearance of the overlapping semi‐transparent points. Labels show Pearson correlation coefficients (*R*) and *p*‐values. Regression slopes are constrained by the number of compounds per group and should only be compared between equally sized groups. For clarity, the data from the four bogs is pooled together.
**Figure S9:** Number of significant effects in *Mychonastes* strain P4 from the Elbe estuary with and without addition of ampicillin. Significant effects (*p* ≤ 0.05) were determined using one‐sided Welch's *t*‐tests with 3 replicates for both compounds and controls. Points show the different included plates which cover different growth phases, indicated by shape. Differences between the two treatments groups ‘with ampicillin’ and ‘without ampicillin’ were not significant (*p* = 0.783) according to a two‐sided Welch's *t*‐test. Mean values are 18 (without ampicillin) and 17 (with ampicillin). Details see publication 10.1098/rspb.2023.2713.

**Table S1:** Metadata.
**Table S2:** Substrate data.
**Table S3:** Values Figures 3a + 4a.
**Table S4:** Values Figures 3b + 4b.
**Table S5:** Results per plate.
**Table S6:** Results per plate + compound.
**Table S7:** GLMMs.
**Table S8:** Map data for Figure 5.
**Table S9:** Data.

## Data Availability

The Supporting Information shows the raw measurement values (e.g., cell counts; Table S9), all numeric results referred to in the manuscript (e.g., *p*‐values, effect sizes; Tables S3–S7) as well as metadata (Table S1). Further raw data and metadata have been published elsewhere for the Elbe estuary (https://doi.org/10.25592/uhhfdm.18666), the Baltic Sea (https://zenodo.org/records/3958784) and the Mediterranean Sea (https://doi.org/10.25592/uhhfdm.18670).
