# Supplementary material for: Osmomixotrophy in Phytoplankton: Analysis of 46 Strains From Marine and Freshwater Habitats Indicates Prevalent Use of Organic Compounds
Source: Environ Microbiol Rep. 2026 Sep 23;18(5):e70420. doi: 10.1111/1758-2229.70420 (PMC13601737; doi:10.1111/1758-2229.70420)
Supplement: Supplementary file 1 [file EMI4-18-e70420-s002.pdf]

Supplementary figures

Osmomixotrophy in Phytoplankton: Analysis of 46 Strains from Marine and Freshwater Habitats Indicates Prevalent Use of Organic Compounds

Nele Martens, Luisa Listmann, Jette Ludewigs, C.-Elisa Schaum

Fig. S1: Significant effects (green) of each compound on each ecoplate.

Significant effects (p ≤ 0.05) were determined using one-sided Welch's t-tests with 3 replicates per compound and 2 - 3 replicates per control. The horizontal scale shows the different compounds (bottom) sorted by compound group (top). On the vertical axis we show the different plates which include different taxa, strains, incubation times, temperatures, growth phases and replicates. Precisely, it is named as such: >>taxon – strain – temperature – growth phase (1 – 3 = early - late) – replicate<<. The vertical panels show different ecosystems (BS = Baltic Sea, MS = Mediterranean Sea, EE = Elbe estuary, Bo = Bogs) and functional groups (Des = desmids, Chry = chrysophytes, PG = pico green algae, NG = nano green algae, Dia = diatoms, Cya = cyanobacteria) (see also Tab. S1). For the bog desmids, different origin is indicated (ZL = Zeller Loch, VL = Puddly near Vlasina Lake, WW = Wohldorfer Wald, CX = Cuxhaven).

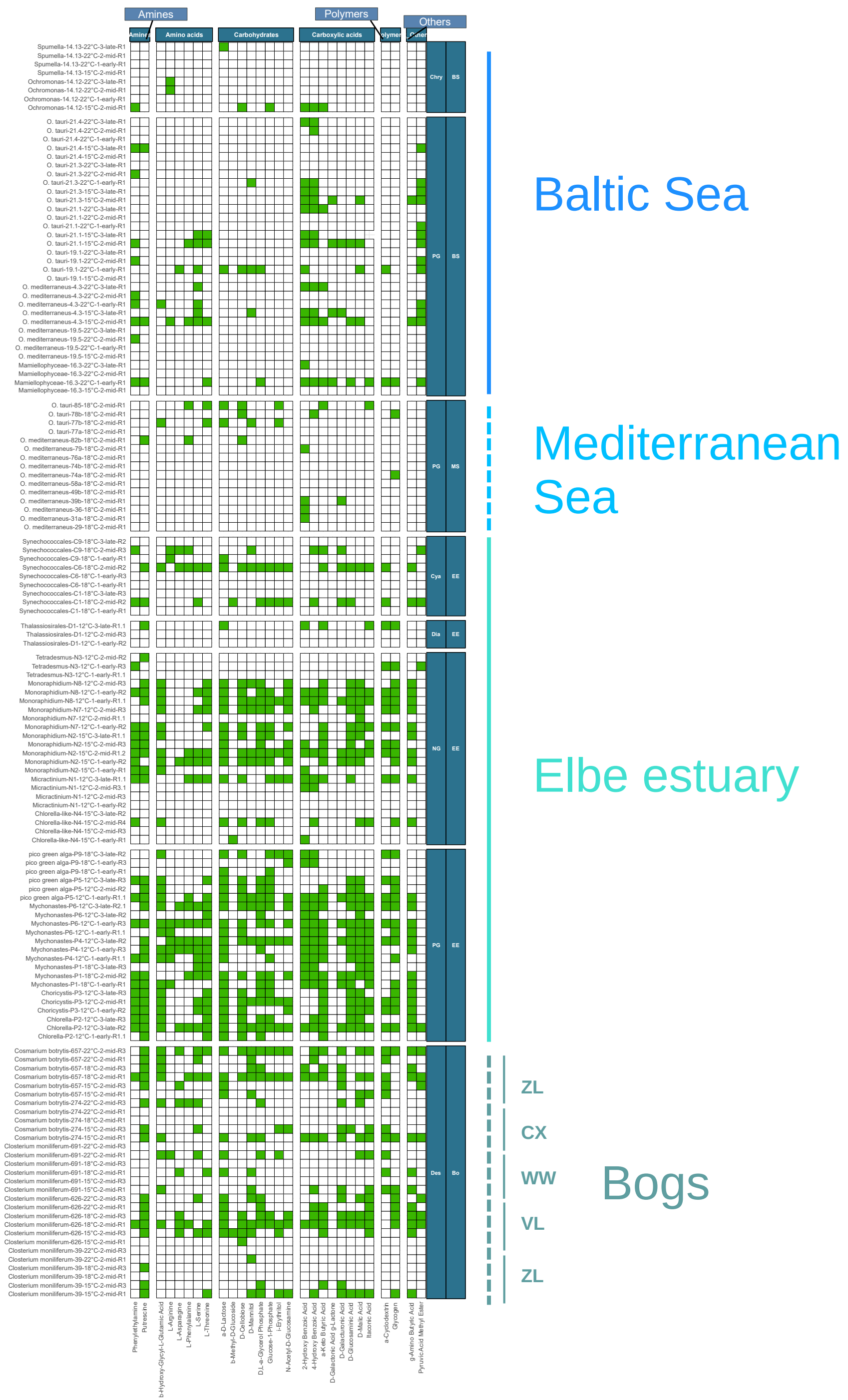

green = significant effect

white = no significant effect

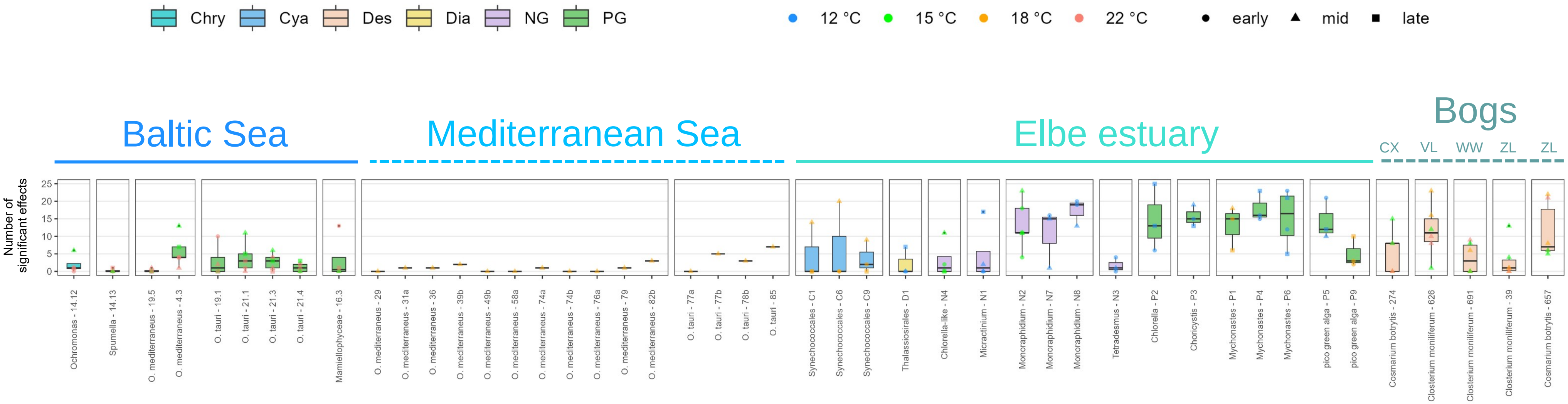

Fig. S2: Number of significant effects per ecoplate clustered by strains covering different taxa from different habitats. Significant effects ($p \leq 0.05$) were determined using one-sided Welch's t-tests with 3 replicates per compound and 2 - 3 replicates per control. Color scheme of the boxplots indicates functional group (Des = desmids, Chry = chrysophytes, PG = pico green algae, NG = nano green algae, Dia = diatoms, Cya = cyanobacteria). Color scheme of the points indicates the experimental temperature. Point shape shows the timepoint during the exponential phase. Note that effect size was not obtained for the bog dataset, explaining the absence of '+' here. For the bog desmids, different origin is indicated (ZL = Zeller Loch, VL = Puddly near Vlasina Lake, WW = Wohldorfer Wald, CX = Cuxhaven).

Fig. S3: Effect sizes of each compound with significant effects per ecoplate (means of well triplicates; SD see Tab. S6).

Significant effects (p ≤ 0.05) were determined using one-sided Welch's t-tests with 3 replicates per compound and 2 - 3 replicates per control. Effect size shows how much higher the measurement value (cell count, fluorescence) was in the presence of organic compounds compared to the control [%]. The horizontal scale shows the different compounds (bottom) sorted by compound group (top). On the vertical axis we show the different plates which include different taxa, strains, incubation times, temperatures, growth phases and replicates. Precisely, it is named as such: >>taxon – strain – temperature – growth phase (1 – 3 = early - late) – replicate<<. The vertical panels show different ecosystems (BS = Baltic Sea, MS = Mediterranean Sea, EE = Elbe estuary) and functional groups (Chry = chrysophytes, PG = pico green algae, NG = nano green algae, Dia = diatoms, Cya = cyanobacteria) (see also Tab. S1). Effect size was not obtained for the bog dataset.

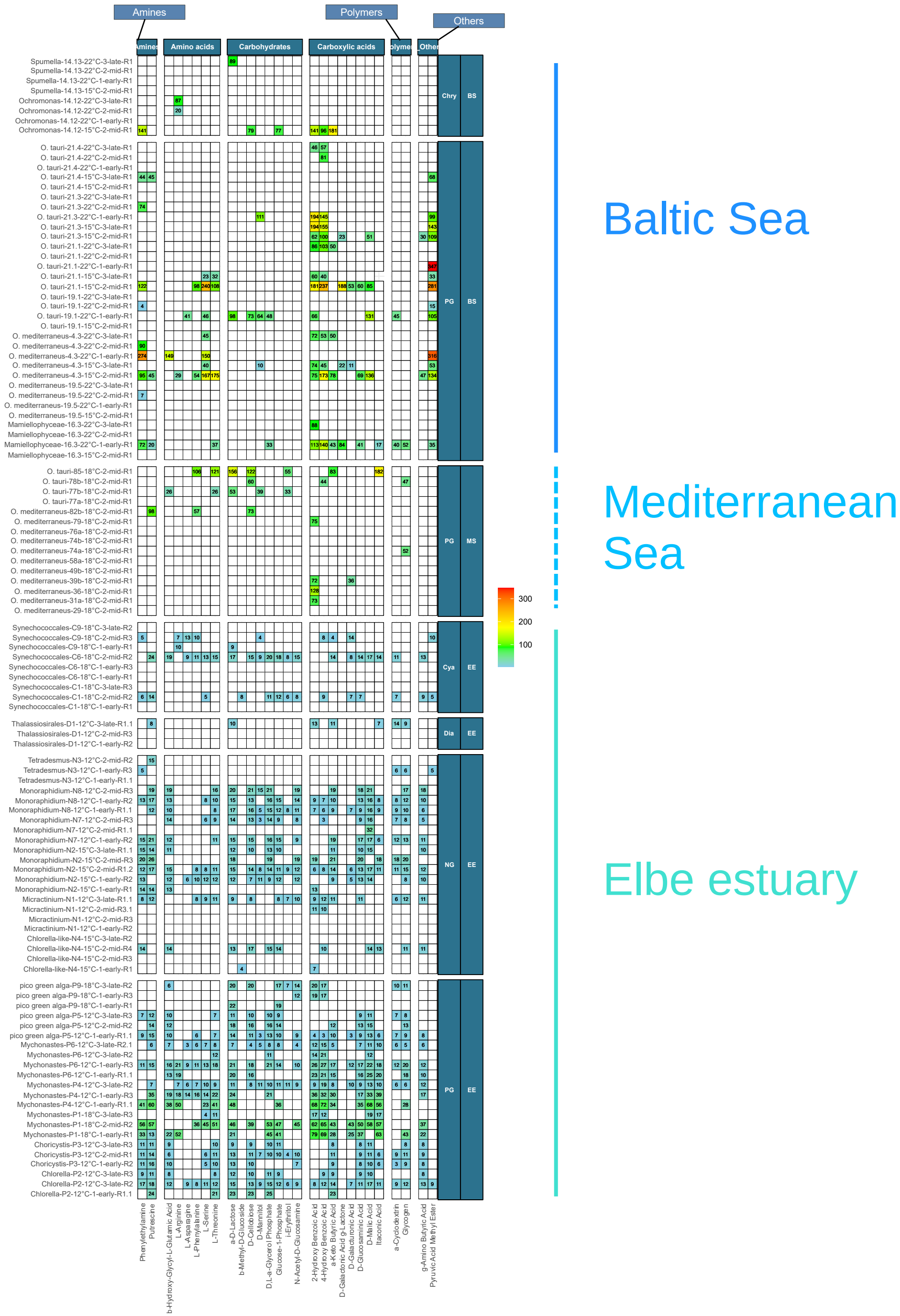

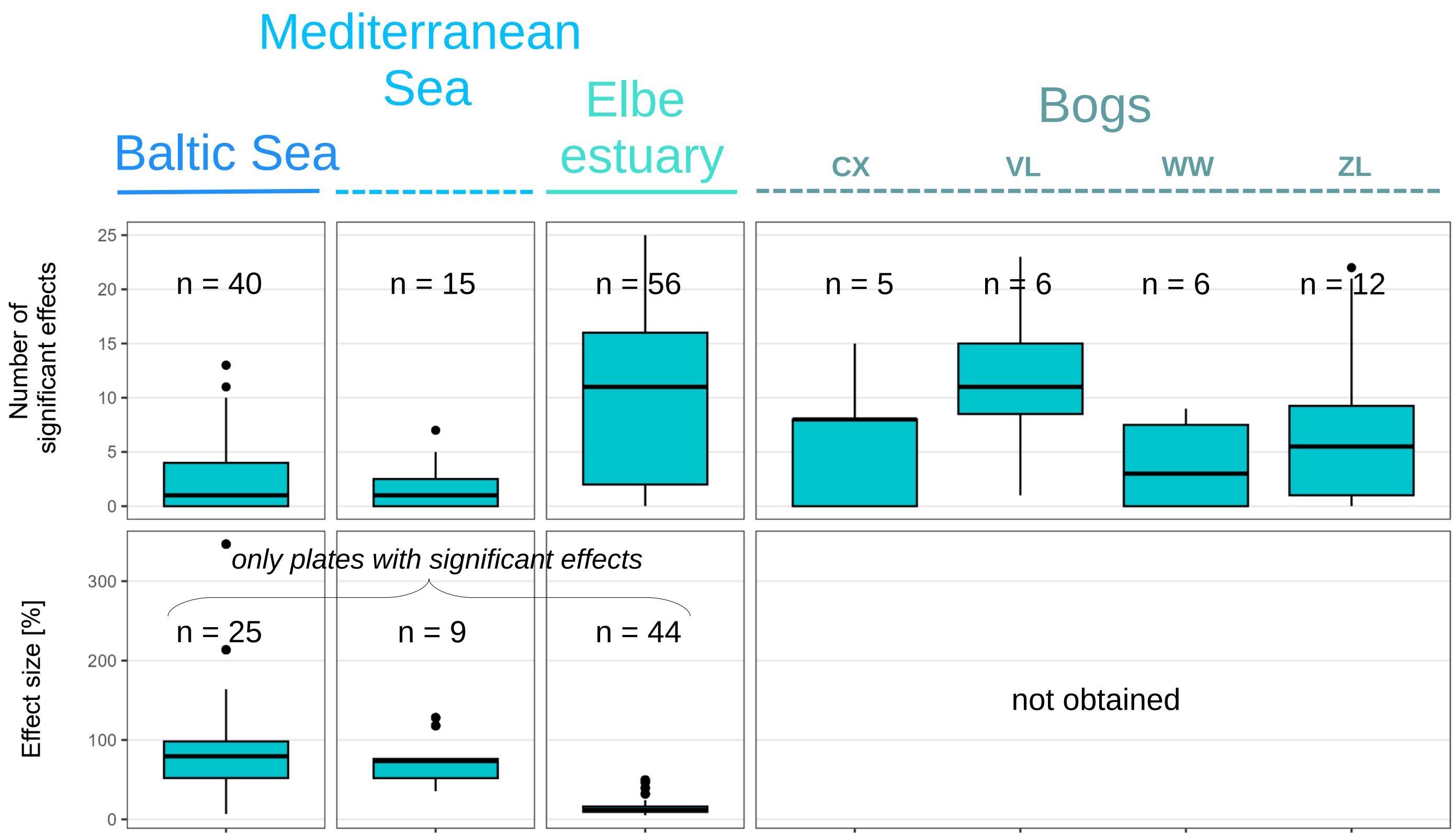

Fig. S4: Number of significant effects per plate and average effect size per plate clustered by habitat. We here show the data across all plates (n = 140) including different taxa, temperatures, growth phases per habitat (for details see also e.g. Tab. S5, Fig. S1 - S3). Significant effects ($p \leq 0.05$) were determined using one-sided Welch's t-tests with 3 replicates per compound and 2 - 3 replicates per control. Effect size shows how much higher the measurement value (cell count, fluorescence) was in the presence of organic compounds compared to the control. ZL = Zeller Loch, VL = Puddly near Vlasina Lake, WW = Wohldorfer Wald, CX = Cuxhaven. Note that effect size was not obtained for the bog dataset.

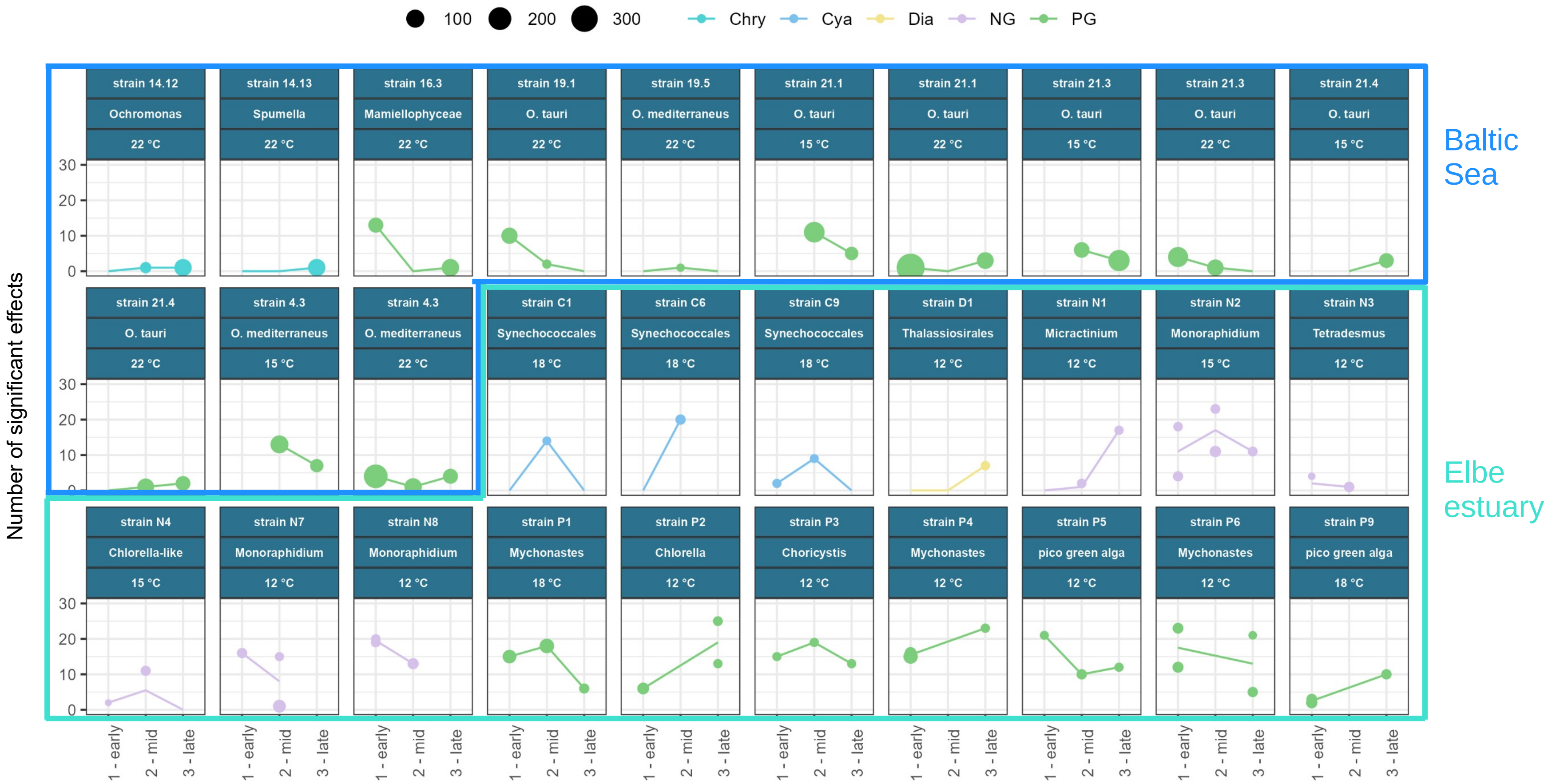

Fig. S5: Number of significant effects along growth phases (1 = early, 2 = mid, 3 = late exponential). Significant effects ($p \leq 0.05$) were determined using one-sided Welch's t-tests with 3 replicates per compound and 2 - 3 replicates per control. Panels show the different taxa, strains (e.g. 16.3) and temperatures. Color scheme indicates functional group (Chry = chrysophytes, PG = pico green algae, NG = nano green algae, Dia = diatoms, Cya = cyanobacteria). Data points are connected by a simple line as a visual aid. Point size shows the mean effect size of significant effects per plate [%] as indicated above (no significant effects = no point). Where more than one bioreplicate was included, line is drawn through the mean. Strain and temperature combinations that did not cover multiple growth phases were removed, which also concerns the complete bog and Mediterranean Sea data.

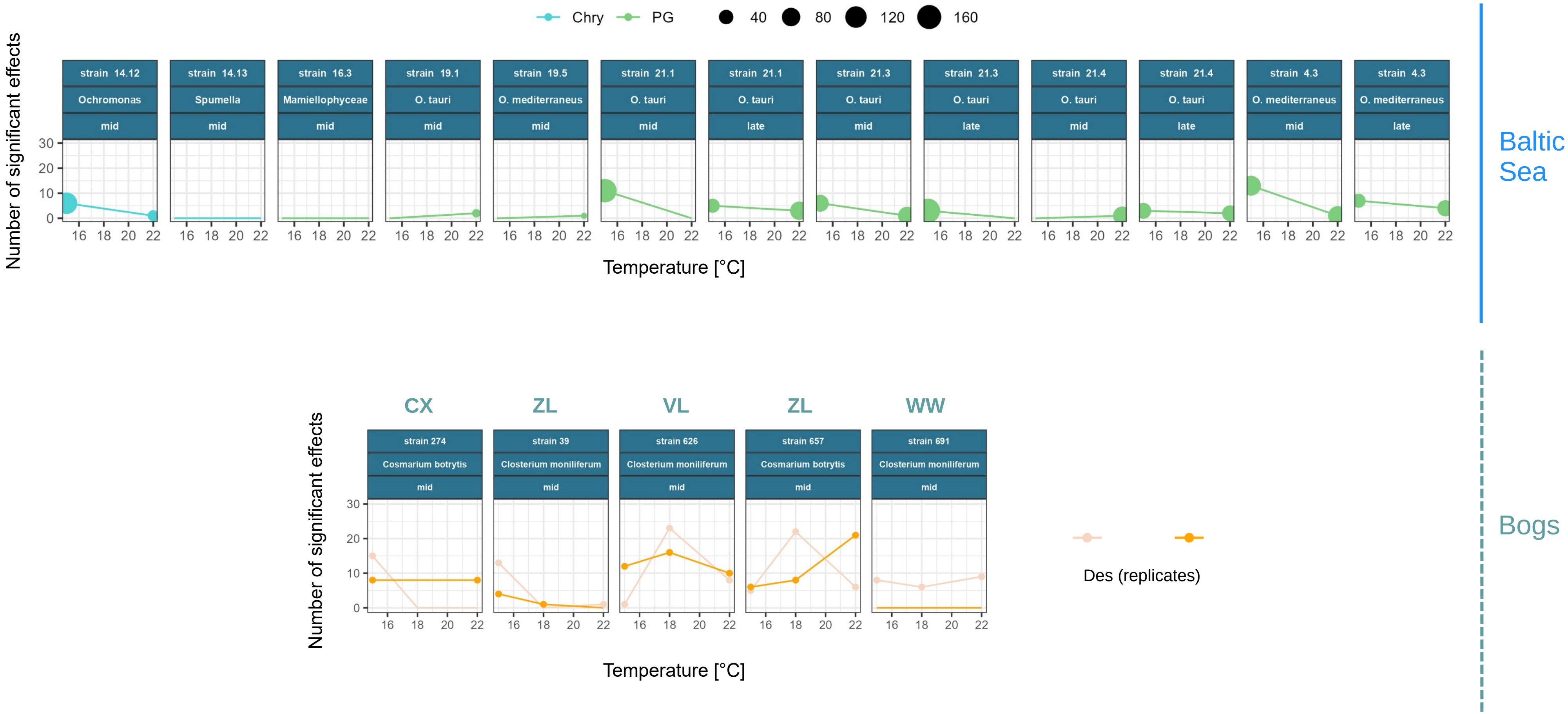

Fig. S6: Number of significant effects along temperatures. Significant effects ($p \leq 0.05$) were determined using one-sided Welch's t-tests with 3 replicates per compound and 2 replicates per control. Panels show the different taxa, strains (e.g. 14.12) and growth phases. Colors indicate the functional group (Chry = chrysophytes, PG = pico green algae, Des = desmids), and for the desmids also different bioreplicates. Data points are connected by a simple line as a visual aid. For the Baltic Sea data point size shows the mean effect size of significant effects per plate [%] as indicated above. For the bog data point size is fixed where effects appeared, as effect size was not obtained. In both datasets, no point indicates no significant effects (i.e. y = 0). Note that strain 274 has only 2 data points in one of the replicates (15 °C and 22 °C). Strain and growth phase combinations that did not cover multiple temperatures were removed, which also concerns the complete Elbe estuary and Mediterranean Sea data. For the bog desmids, different origin is indicated (ZL = Zeller Loch, VL = Puddly near Vlasina Lake, WW = Wohldorfer Wald, CX = Cuxhaven).

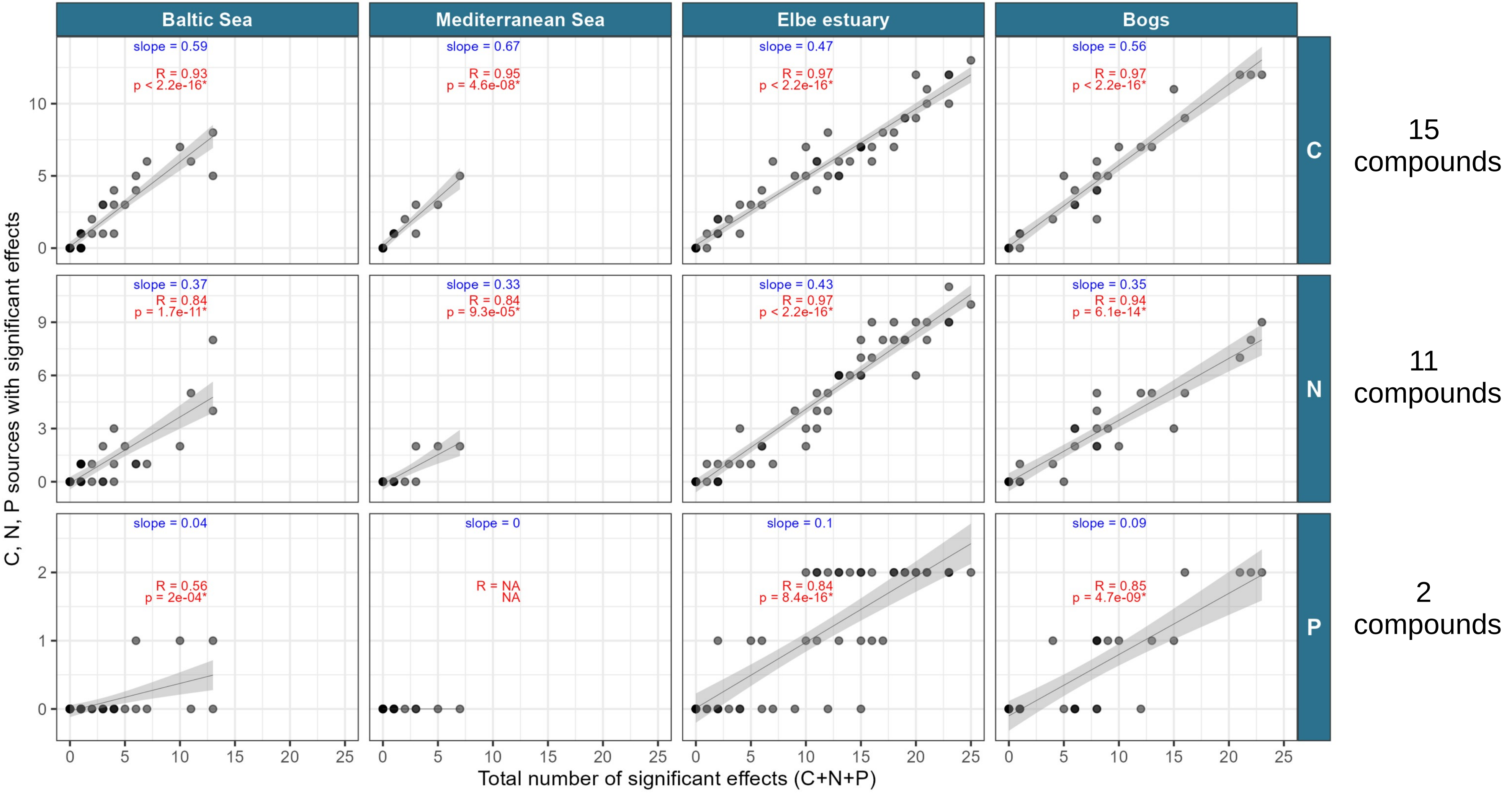

Fig. S7: C, N and P sources with significant effects per plate, plotted against the total number of significant effects on each plate (across all 140 included plates; per habitat). Significant effects ($p \le 0.05$) were determined using one-sided Welch's t-tests with 3 replicates per compound and 2 - 3 replicates per control. Note that plates contained 2 P sources, 11 N sources and 15 sole C sources. Data points partially overlap. This is visible by darker appearance of the overlapping semi-transparent points. Labels show Pearson correlation coefficients (R) and p-values. Note: Regression slopes are constrained by the number of compounds per group and should only be compared between equally sized groups. For clarity, the data from the four bogs is pooled together.

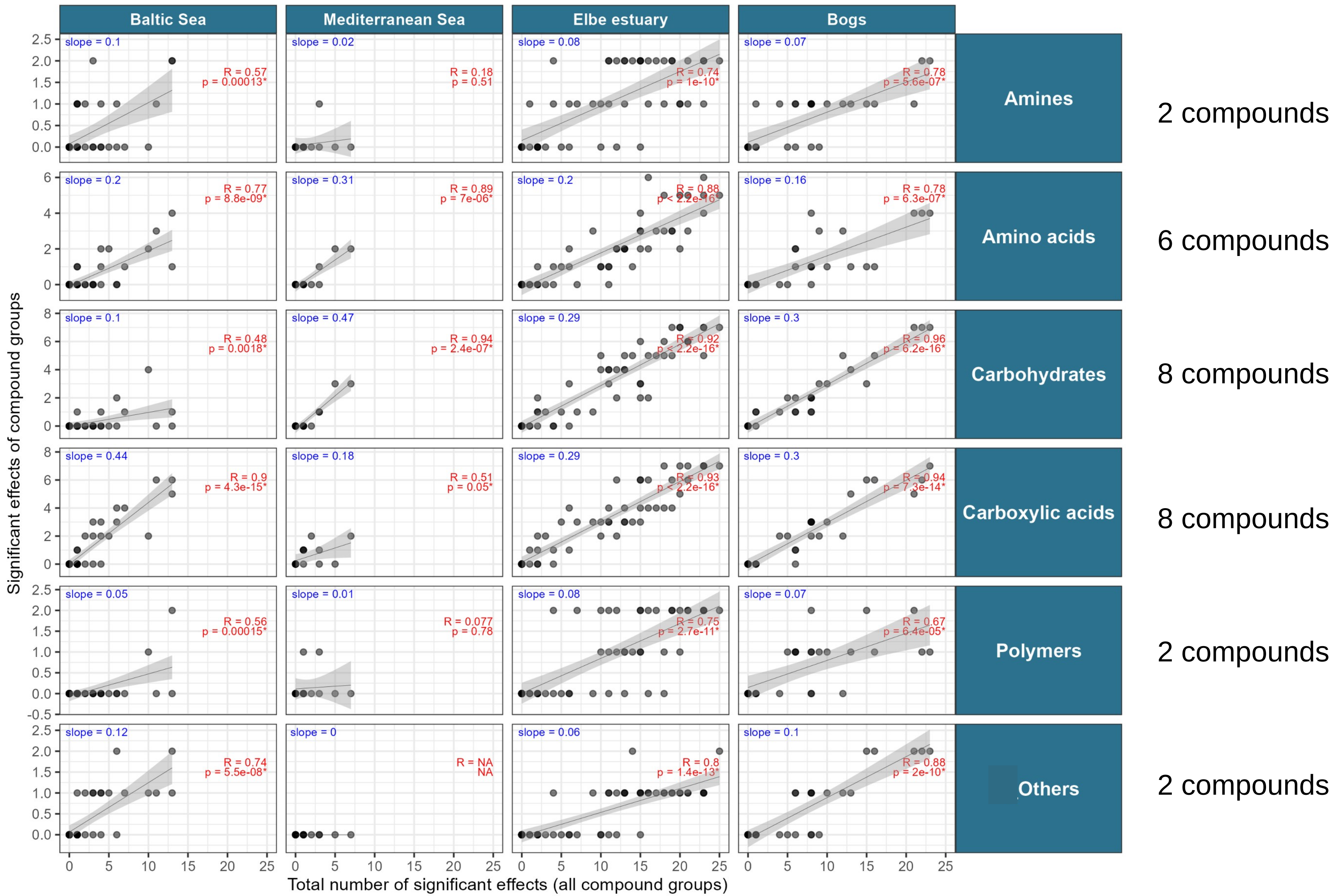

Fig. S8: Compounds belonging to the different compound groups with significant effects per plate, plotted against the total number of significant effects on each plate (across all 140 included plates; per habitat). Significant effects ($p \leq 0.05$) were determined using one-sided Welch's t-tests with 3 replicates per compound and 2 - 3 replicates per control. Note that plates contained 2 amines, 6 amino acids, 8 carbohydrates, 8 carboxylic acids, 2 polymers, and 2 other compounds. Data points partially overlap. This is visible by darker appearance of the overlapping semi-transparent points. Labels show Pearson correlation coefficients (R) and p-values. Note: Regression slopes are constrained by the number of compounds per group and should only be compared between equally sized groups. For clarity, the data from the four bogs is pooled together.

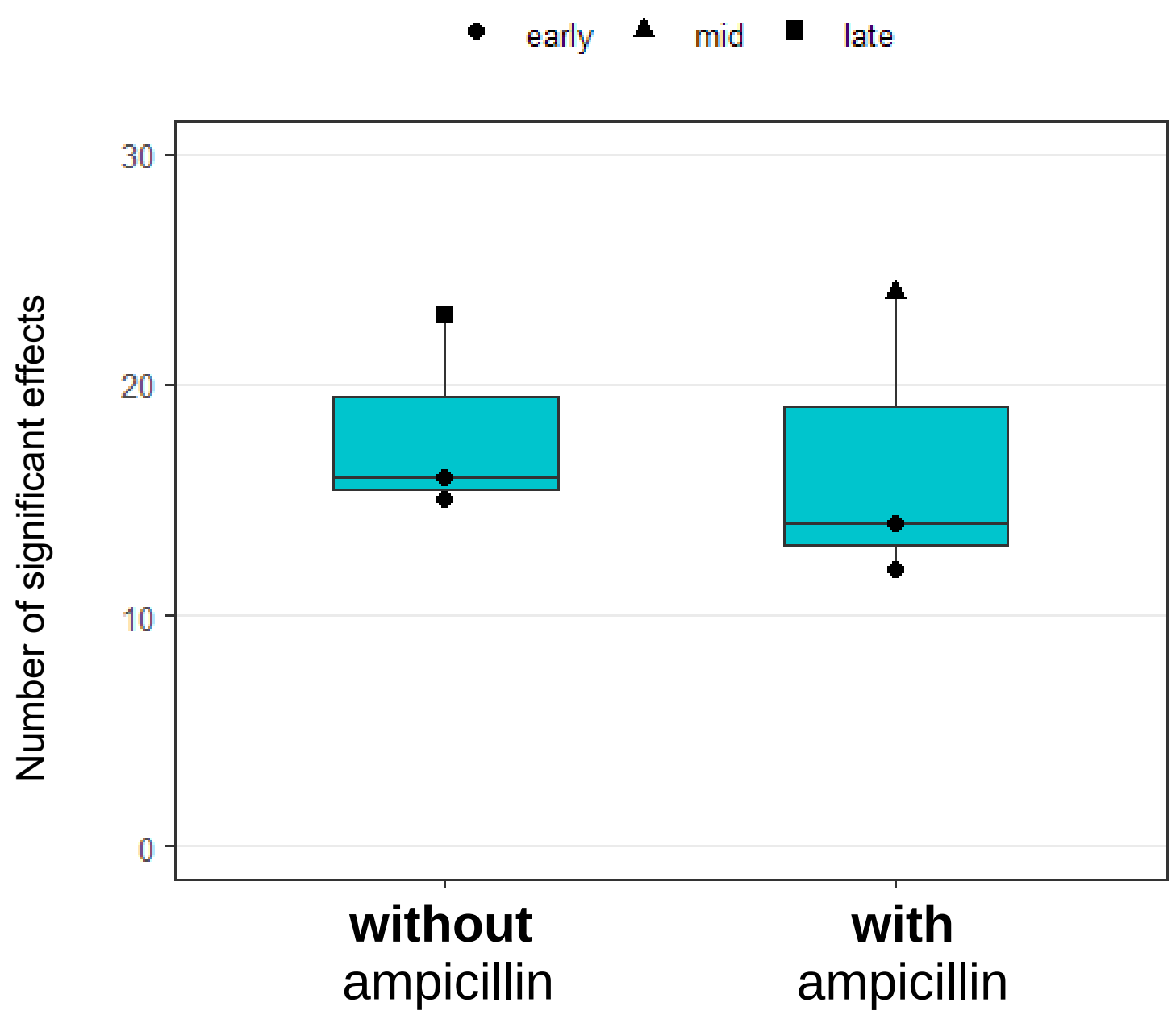

Fig. S9: Number of significant effects in *Mychonastes* strain P4 from the Elbe estuary with and without addition of ampicillin. Significant effects ($p \leq 0.05$) were determined using one-sided Welch's t-tests with 3 replicates for both compounds and controls. Points show the different included plates which cover different growth phases, indicated by shape. Differences between the two treatments groups "with ampicillin" and "without ampicillin" were not significant ($p = 0.783$) according to a two-sided Welch's t-test. Mean values are 18 (without ampicillin) and 17 (with ampicillin). Details see publication 10.1098/rspb.2023.2713.
